# Liraglutide Promotes Diabetic Wound Healing via Myo1c/Dock5

**DOI:** 10.1002/advs.202405987

**Published:** 2024-08-19

**Authors:** Qian Zhang, Chunlin Zhang, Changjiang Kang, Jiaran Zhu, Qingshan He, Hongwei Li, Qiang Tong, Min Wang, Linlin Zhang, Xin Xiong, Yuren Wang, Hua Qu, Hongting Zheng, Yi Zheng

**Affiliations:** ^1^ School of Life Sciences Chongqing University Chongqing 401331 China; ^2^ Department of Endocrinology Translational Research of Diabetes Key Laboratory of Chongqing Education Commission of China the Second Affiliated Hospital of Army Medical University Chongqing 400037 China; ^3^ Department of Pharmacy the Second Affiliated Hospital of Army Medical University Chongqing 400037 China; ^4^ Department of Laboratory Medicine Chongqing University Three Gorges Hospital School of Medicine Chongqing University Chongqing 404000 China; ^5^ Department of Medicinal Chemistry Army Medical University Chongqing 400038 China

**Keywords:** diabetic wound healing, Dock5, keratinocyte function, liraglutide

## Abstract

Non‐healing diabetic wounds and ulcer complications, with persistent cell dysfunction and obstructed cellular processes, are leading causes of disability and death in patients with diabetes. Currently, there is a lack of guideline‐recommended hypoglycemic drugs in clinical practice, likely due to limited research and unclear mechanisms. In this study, it is demonstrated that liraglutide significantly accelerates wound closure in diabetic mouse models (db/db mice and streptozotocin‐induced mice) by improving re‐epithelialization, collagen deposition, and extracellular matrix remodeling, and enhancing the proliferation, migration, and adhesion functions of keratinocytes. However, these effects of improved healing by liraglutide are abrogated in dedicator of cytokinesis 5 (Dock5) keratinocyte‐specific knockout mice. Mechanistically, liraglutide induces cellular function through stabilization of unconventional myosin 1c (Myo1c). Liraglutide directly binds to Myo1c at arginine 93, enhancing the Myo1c/Dock5 interaction by targeting Dock5 promoter and thus promoting the proliferation, migration, and adhesion of keratinocytes. Therefore, this study provides insights into liraglutide biology and suggests it may be an effective treatment for diabetic patients with wound‐healing pathologies.

## Graphical abstract

Current study demonstrates that liraglutide significantly accelerates wound closure in diabetic mouse models by improving re‐epithelialization, collagen deposition, and extracellular matrix remodeling, and enhancing the proliferation, migration, and adhesion functions of keratinocytes. Mechanically, liraglutide directly binds to Myo1c at arginine 93, enhancing the Myo1c/Dock5 interaction by targeting Dock5 promoter and thus promoting the functions of keratinocytes. This study suggests liraglutide may serve the treatment of diabetic wound healing.

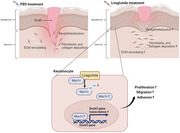

## 1 Introduction

Diabetic foot ulcers (DFU) and wounds are serious and common complications of diabetes, with an incidence of ≈30% among diabetic patients.^[^ ^1^ ^]^ DFUs are associated with increased rates of death and amputation; for example, the 5‐year mortality rate is up to 30% and >70% for patients with DFU undergoing major amputation.^[^ ^2^ ^]^ Currently, clinical guidelines recommend DFU as standard care, which consists of surgical debridement, reduction of pressure from weight‐bearing, and treatment of lower extremity ischemia and foot infection.^[^ ^3^, ^4^ ^]^ Good blood sugar management is generally considered to reduce the risk of complications in patients with diabetes,^[^ ^5^ ^]^ however, clinical guidelines for recommended hypoglycemic drugs have not been established to date. In contrast, sodium‐dependent glucose transporter 2 inhibitors have been previously reported to be associated with an increased risk of amputation in patients with diabetes.^[^ ^6^, ^7^ ^]^ Under these circumstances, insulin is commonly used in clinical practice,^[^ ^8^ ^]^ but its appropriateness is uncertain due to the lack of data on the efficacy of individual glucose‐lowering agents in diabetic wounds.

Some related research is being conducted. For example, a previous study using the existing data from the LEADER (Liraglutide Effect and Action in Diabetes: Evaluation of Cardiovascular Outcome Results) trial, where DFU was a pre‐specified secondary endpoint,^[^ ^9^ ^]^ found that liraglutide was associated with a significantly lower risk of DFU‐related amputations.^[^ ^10^ ^]^ However, this correlation could also be a result of chance and requires further investigation. More importantly, although liraglutide shows a promoting effect, the underlying mechanisms of diabetic wound healing remain unclear. In this study, we found that liraglutide enhanced keratinocyte function and promoted wound healing in diabetic mice through dedicator of cytokinesis 5 (Dock5) signaling. Liraglutide binds to unconventional myosin 1c (Myo1c) to mediate the upregulation of Dock5, thereby improving its downstream effects on keratinocytes. Therefore, liraglutide may have additional benefits in the treatment of DFU and potential clinical applications.

## 2 Result

### 2.1 Liraglutide Promotes keratinocyte Functions and Accelerates Diabetic Wound Healing

Mounting evidence suggests that targeting keratinocytes may be an effective strategy for discovering new treatment strategies for skin diseases such as psoriasis and epidermal wound repair.^[^ ^11^, ^12^, ^13^, ^14^ ^]^ The physiological characteristics of keratinocytes, including proliferation, migration, and adhesion, are vital for cutaneous wound healing.^[^ ^15^, ^16^, ^17^ ^]^ Therefore, we first examined the effects of liraglutide and insulin on keratinocyte function. As shown in Figure S1 (Supporting Information), insulin promoted cell migration as assessed by the wound scratching assay, and liraglutide increased the rate of proliferation, migration, and accelerated adhesion compared to the control. These data prompted us to explore the potential contribution of the hypoglycemic drug liraglutide to the process of wound healing.

**Figure 1:**
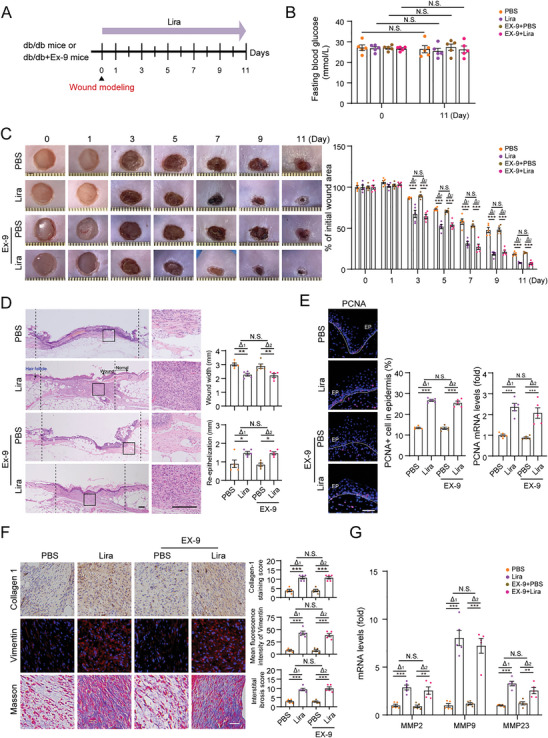
Liraglutide improves wound healing in diabetic db/db mice. Full thickness wounds were made on the dorsal skin of db/db mice, and liraglutide and the GLP‐1R antagonist exendin (9‐39) (Ex‐9) was subcutaneously injected around the wound margins as indicated. A) Schematic for in vivo experiments in db/db mice. B) Fasting blood glucose (mmol L^−1^) was measured at day 0 and day 11 after injury. C) Images of representative wound (left) and the percentage of the initial wound area (right). Each grid scale represents 1 mm. D) H&E of dorsal skin section was showed and the wound width and neoepithelial length were quantified after 7 days post‐injury. Scale bar = 200 µm. E) Representative images and the percentage of PCNA‐positive cells in epidermis, and the PCNA mRNA was analyzed by qRT‐PCR in wound tissues. EP: epidermis. Scale bar = 50 µm. F) Representative immunostaining images of collagen 1, vimentin and Masson's trichrome and their quantifications were showed. Scale bar = 50 µm. G) The mRNA levels of indicated genes were analyzed by qRT‐PCR in wound tissues. n = 5 mice per group for B‐G. Data are expressed as means ± S.E.M. Statistical analyses were performed using one‐way or two‐way ANOVA test with Student's *t* test for B‐G. *P < 0.05, **P < 0.01, ***P < 0.001, N.S. not significant.

Next, the effects of liraglutide on diabetic wound healing were investigated in db/db mice. Moreover, the GLP‐1 receptor (GLP‐1R) antagonist exendin (9‐39) (EX‐9) was applied along with liraglutide to examine the involvement of GLP‐1R, although the skin is not the main organ expressing GLP‐1R, with even lower expression in keratinocytes (https://www.proteinatlas.org/). Two full‐thickness wounds were made on the dorsal side of the mice, and liraglutide was subcutaneously injected around the wound margins (**Figure** **1**A). There were no significant changes of fasting blood glucose (FBG) across all measured time points (Figure 1B; Figure S2, Supporting Information). Liraglutide significantly accelerated the wound closure rate, as assessed by measuring the wound area at the indicated time points (Figure 1C). Re‐epithelialization, measured by the wound width and length of the neoepithelium, is an important process of repair.^[^ ^18^ ^]^ Hematoxylin and eosin (H&E) staining showed shorter wound widths and longer neoepithelial lengths in liraglutide treated db/db mice (Figure 1D), indicating increased re‐epithelialization. Consistently, liraglutide increased PCNA expression in the wounded skin (Figure 1E), suggesting increased proliferation. Concurrently with re‐epithelialization during wound healing, new extracellular matrix (ECM) proteins, such as collagen, is following deposited and granulation tissue is formed.^[^ ^17^, ^18^ ^]^ Immunostaining assay showed that liraglutide increased collagen 1 and the granulation tissue marker vimentin in diabetic wound skin, and elevated collagen generation in granulation tissue was detected by Masson's trichrome staining (Figure 1F). Matrix metalloproteinases (MMPs) are responsible for ECM remodeling in helping to contract the wound.^[^ ^18^, ^19^ ^]^ The upregulated expression of MMP2, MMP9, and MMP23 in liraglutide‐treated mice was assessed (Figure 1G). In addition, other classical documentation of wound healing, such as inflammation and angiogenesis, were also observed to undergo changes after treatment with liraglutide (Figure S3, Supporting Information). More importantly, the accelerated wound closure observed in liraglutide treated db/db mice, including re‐epithelialization, granulation formation, ECM remodeling, inflammation and angiogenesis, were not altered by administration of EX‐9 (Figure 1B–G; Figure S3, Supporting Information). These findings demonstrated that liraglutide‐induced diabetic wound healing may not primarily through GLP‐1R signaling. We also evaluated the functions of keratinocytes when GLP‐1R was knocked down in vitro. GLP‐1R knockdown (si‐GLP‐1R) successfully inhibited the activation of GLP‐1R signaling (indicated by the production of cyclic adenosine monophosphate (cAMP), an indicator of GLP‐1R activation^[^ ^20^, ^21^ ^]^ (Figure S4A, Supporting Information). However, liraglutide promoted keratinocyte behaviors remained even after knocking down GLP‐1R in these cells (Figure S4B–D, Supporting Information).

Liraglutide has also been reported to improve glycemia and reduce adiposity in type 1 diabetes.^[^ ^22^, ^23^ ^]^ Thus, we employed streptozotocin (STZ)‐induced diabetic mice to confirm the impact of liraglutide. In accordance with the data observed in db/db mice, liraglutide improved re‐epithelialization, collagen deposition, and ECM remodeling in STZ mice (Figure S5A–G, Supporting Information).

### 2.2 Liraglutide Promotes Wound Healing through Dock5 Signaling

**Figure 2:**
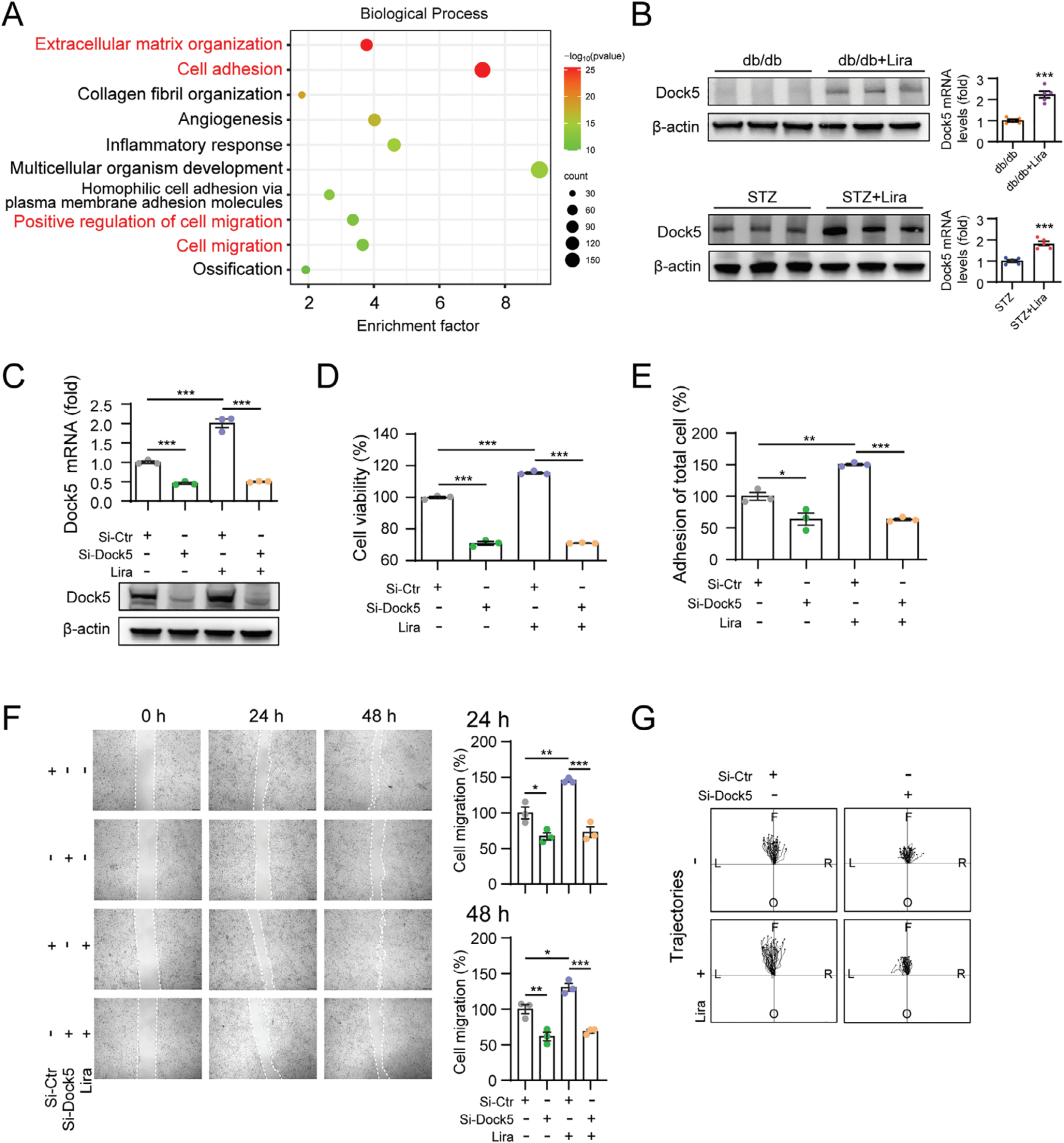
Liraglutide facilitates proliferation, adhesion and migration of keratinocyte through Dock5. A) The top 10 items of Biological Process from gene ontology analysis of upregulated DEGs *P* < 0.05, fold change > 1.5 using wound skin tissues of db/db mice treated with or without liraglutide. B) Dock5 protein and mRNA levels in wound skin tissues from db/db+Lira and STZ+Lira mice. C–G) Keratinocytes were transfected with siRNA targeting Dock5, and treated with or without liraglutide. C) The mRNA and protein expression of Dock5 were assessed. D) Cell proliferation was evaluated by Cell Counting Kit 8 assay. E) Cell adhesion was determined using the CyQUANT Assay Kit. F) The migratory ability at indicated time was evaluated with wound scratching assays. G) Live cell imaging‐based migration assay was used to track cell migration trajectory after scratching. The center represents starting point, and black dots represent the ending point. L: left, R: right, F: forward, O: opposite. n = 3 mice per group for A. n = 5 mice per group for B. n = 3 for C‐F. n = 40 for G. Data are expressed as means ± S.E.M. Statistical analyses were performed using Student's *t* test for B, one‐way ANOVA test for C‐F. *P < 0.05, **P < 0.01, ***P < 0.001, N.S. not significant.

Next, we performed RNA sequencing analysis of liraglutide‐treated wound skin samples from db/db mice (PBS‐treated wound skin was used as a control) to explore the potential mechanisms. Biological Process analysis showed that gene ontology (GO) terms of upregulated differentially expressed genes (DEGs) were mainly associated with “extracellular matrix”, “cell adhesion” and “cell migration” pathways (**Figure** **2**A; Table S1, Supporting Information). Our research team has previously revealed the role of Dock5 in promoting wound healing by regulating keratinocyte adhesion, migration, and proliferation, and influencing the functions of ECM deposition,^[^ ^24^ ^]^ which was in line with the current observations of liraglutide. We evaluated whether Dock5 serves as a mediator of liraglutide stimulation. First, both the protein and mRNA levels of Dock5 were increased in the wounded skin tissues of liraglutide‐treated db/db and STZ mice (Figure 2B). We then knocked down Dock5 to examine its role in liraglutide‐regulated keratinocyte function (Figure 2C). The results showed that inhibition of Dock5 abolished liraglutide‐induced proliferation, adhesion, and migration, as assessed by both wound scratching and live cell imaging‐based migration assays (Figure 2D–G), suggesting that Dock5 may be involved in liraglutide‐enhanced keratinocyte functions.

**Figure 3:**
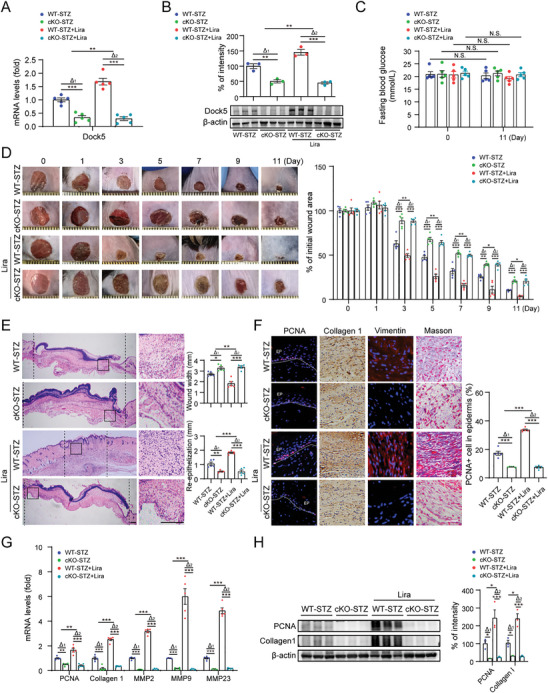
Dock5 deletion in keratinocytes abolishes liraglutide‐induced wound healing in diabetic mice. Full thickness wounds were made on the dorsal skin of STZ‐injected WT mice (WT‐STZ) and Dock5 cKO mice (cKO‐STZ), and liraglutide was subcutaneously injected around the wound margins once a day after the wounds were created. A) Dock5 mRNA and B) protein levels and their quantification in wound skin tissue. C) Fasting blood glucose (mmol L^−1^) was assessed at day 0 and day 11 after injury. D) Images of representative wound (left) and the percentage of the initial wound area (right). Each grid scale represents 1 mm. E) H&E of dorsal skin section showing wound width and re‐epithelialization quantified 7 days post‐injury. Scale bar = 200 µm. F) Representative immunostaining images of PCNA, collagen 1, vimentin and Masson's trichrome (left) and the percentage of PCNA‐positive cells in epidermis (right). EP: epidermis. Scale bar = 50 µm. G) The mRNA levels of indicated genes were analyzed by qRT‐PCR in wound tissues. H) Expression of indicated protein in wound tissue from each group of mice detected by western blot analysis, with the percentage of intensity quantified. n = 5 mice per group for A‐H. Data are expressed as means ± S.E.M. Statistical analyses were performed using one‐way or two‐way ANOVA test with Student's *t* test for A, B, and D‐H, and using Student's *t* test for C. *P < 0.05, **P < 0.01, ***P < 0.001, N.S. not significant.

To validate the role of Dock5 in the mediation of liraglutide in vivo, keratinocyte‐specific Dock5 knockout mice (hereafter referred to as cKO), which were bred Dock5 conditional allele (Dock5^flox/flox^) mice with keratin14‐Cre mice, and their littermate wild‐type (WT) mice were induced to develop diabetes. Liraglutide was subcutaneously injected around the wound margins after the wounds were created once a day. Dock5 deletion was verified using qRT‐PCR and western blotting (**Figure** **3**A,B). FBG showed no statistical significance among all groups, although the WT‐STZ+Lira group showed a decreasing trend compared to the cKO‐STZ+Lira group (Figure 3C). Diabetic cKO mice exhibited a significant delay in wound closure compared with diabetic WT mice, as demonstrated by retarded re‐epithelialization, decreased ECM remodeling, and granulation formation. Importantly, liraglutide no longer promoted wound healing in diabetic cKO mice, although improvements were observed in diabetic WT mice (Figure 3D–H). These results indicated that Dock5 deficiency in keratinocytes abolishes liraglutide‐accelerated diabetic wound healing, suggesting that liraglutide exerts its beneficial effects by modulating Dock5 expression.

### 2.3 Liraglutide Elevates Myo1c Expression to Promote Dock5 Transcription

**Figure 4:**
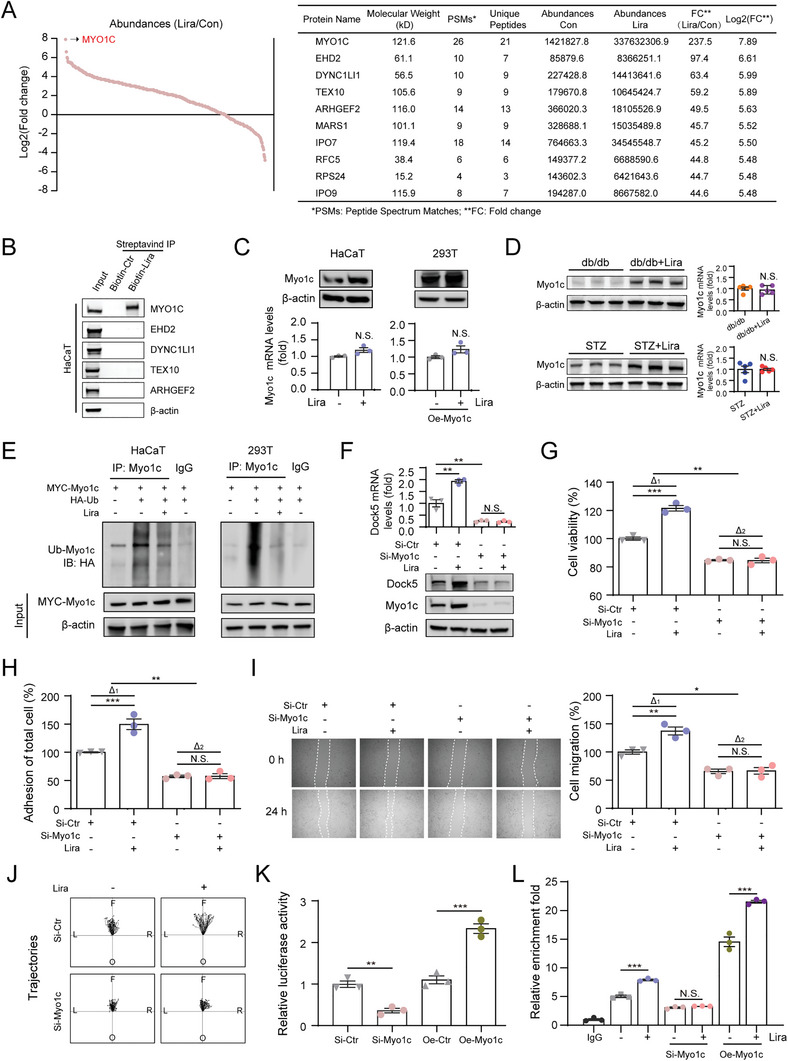
Liraglutide elevates Myo1c expression to promote Dock5 transcription. A) Biotin‐liraglutide pull‐down and mass spectrometry were performed, and all predicted proteins was shown with fold change (left), and the top 10 abundant proteins were characterized (right). B) Biotin‐liraglutide pull‐down assay of the top 5 proteins was performed using HaCaT cell lysates, with biotin‐azide as a control. C and D) Myo1c protein and mRNA levels in liraglutide treated cells C), and in wound skin tissues from db/db+Lira and STZ+Lira mice D). E) Cells were transfected with HA‐tagged ubiquitin (Ub) and MYC‐tagged Myo1c plasmids as indicated, then treated with liraglutide for 24 h and the proteasomal inhibitor MG132 (10 µM) during the last 4 h. The ubiquitination of Myo1c was detected by immunoprecipitation and immunoblotting analysis. F–J) Keratinocytes were transfected with si‐Myo1c and treated with or without liraglutide, and the Dock5 mRNA and protein levels F), cell proliferation G), adhesion H), wound scratching assays I), and live cell imaging‐based migration assay J) were performed. The center represents the starting point, and black dots represent the ending point ending point. L: left, R: right, F: forward, O: opposite. K) Dock5 transcriptional level was determined using luciferase reporter assay. L) Cleavage Under Targets and Tagmentation (CUT&Tag) was performed to examine the binding of Myo1c to Dock5 promoter in keratinocytes. n = 3 for C, F‐I, K and L. n = 40 for J. n = 5 mice per group for D. Data are expressed as means ± S.E.M. Statistical analyses were performed using Student's *t* test for C, D and K, one‐way ANOVA test for F‐I, and L. *P < 0.05, **P < 0.01, ***P < 0.001, N.S. not significant.

To determine the molecular mechanism by which liraglutide regulates Dock5, we performed biotinylated liraglutide (biotin‐Lira) pull‐down assays in keratinocytes.^[^ ^25^ ^]^ The protein bands precipitated by liraglutide, and the matching gel region of the control were stained with Coomassie (Figure S6, Supporting Information) and then cut for mass spectrometry (MS)‐based protein identification. MS identified 524 binding proteins; liraglutide enhanced 418 protein binding and weakened 106 proteins compared to the control (**Figure** **4**A). The top five predicted proteins were subjected to biotinylated liraglutide pull‐down experiments. The results showed that unconventional myosin 1c (Myo1c) was the most abundant and that liraglutide could interact with both endogenous and exogenous Myo1c (Figure 4B; Figure S7, Supporting Information). We also observed increased Myo1c protein levels without changes in mRNA levels in vitro in liraglutide‐treated cells and in vivo in liraglutide‐treated db/db and STZ mice (Figure 4C,D). Furthermore, decreased ubiquitination of Myo1c was confirmed upon liraglutide treatment (Figure 4E), which was consistent with a previous report that proteasome‐regulated degradation of Myo1c,^[^ ^26^ ^]^ indicating that liraglutide stabilizes Myo1c protein by inhibiting its ubiquitination.

Next, we assessed whether Myo1c was responsible for the effects of liraglutide on Dock5 and downstream changes in keratinocytes. The data showed Myo1c‐siRNA infection abrogated liraglutide‐induced Dock5 expression in parallel with cell viability, adhesion, and movement (Figure 4F–J). In contrast, overexpression of Myo1c upregulated Dock5 mRNA and protein levels (Figure S8A, Supporting Information), and favored cell functions, which could not be obtained in si‐Dock5 transfected keratinocyte (Figure S8B–D, Supporting Information). These results indicated that Myo1c may serve as an upstream mediator of liraglutide‐regulated Dock5 expression and keratinocyte behavior.

The aforementioned results indicated that Myo1c influenced Dock5 at both the mRNA and protein levels (Figure 4F; Figure S8A, Supporting Information), and we further addressed the mechanism by which Myo1c regulates Dock5 transcriptional levels. The effect of Myo1c on Dock5 transcriptional activity was determined using a dual‐luciferase reporter assay. The results revealed that Myo1c inhibition (si‐Myo1c) significantly decreased the transcription level of Dock5, whereas overexpression of Myo1c (Oe‐Myo1c) increased Dock5 transcription (Figure 4K). Next, to determine whether Myo1c transcriptionally targets Dock5, we used Cleavage Under Targets and Tagmentation (CUT&Tag) because of its high sensitivity, specificity, and low background levels.^[^ ^27^, ^28^, ^29^ ^]^ Keratinocytes were subjected to CUT&Tag using anti‐Myo1c antibody/IgG antibody, followed by qRT‐PCR of the Dock5 promoter regions. Interestingly, Myo1c enriched the Dock5 promoter by more than five folds when compared to the control IgG, which was enhanced by liraglutide. In Myo1c knockdown cells, liraglutide did not induce enrichment in the binding of the Dock5 promoter; however, the enrichment was further increased by liraglutide under Myo1c overexpression (Figure 4L). These findings further confirm Myo1c promotes the transcription of Dock5 by directly targeting the Dock5 promoter.

### 2.4 Liraglutide Interacts with Myo1c at ARGININE 93

**Figure 5:**
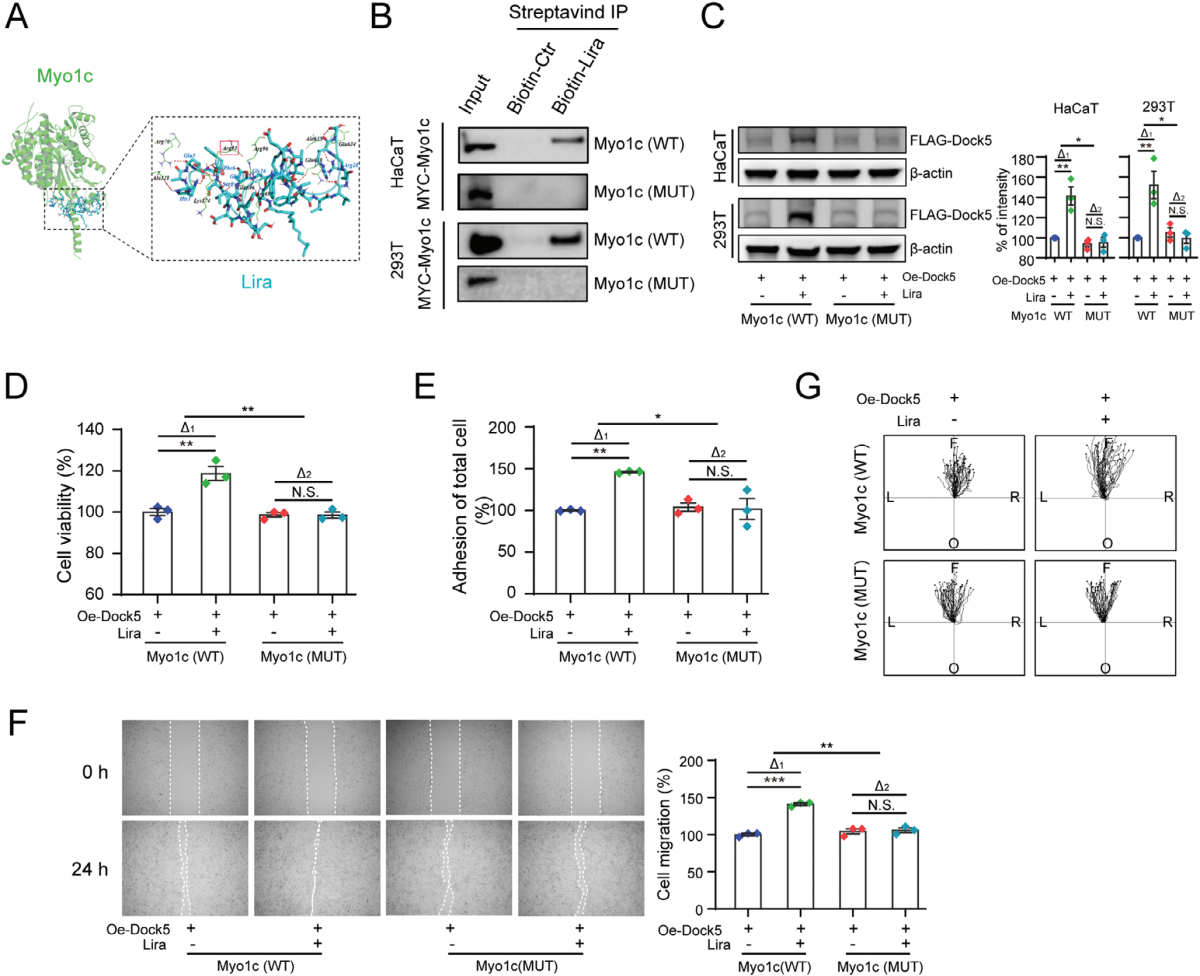
Liraglutide interacts with Myo1c at Arginine 93. A) Molecular docking model of liraglutide interacting with Myo1c (human Myo1c, PDB ID: 4BYF). B) Cells were transfected with MYC‐tagged Myo1c (Myo1c‐WT) and MYC‐tagged Myo1c R93G mutation (Myo1c‐MUT) plasmids. The biotin‐liraglutide pull‐down assay are shown for the indicated cell. C–G) Cells were respectively transfected with Myo1c‐WT and Myo1c‐MUT with FLAG‐tagged Dock5 plasmids, and western blot to detect exogenous Dock5 C), cell proliferation D), adhesion E), wound scratching assays F), and live cell imaging‐based migration assay G) were assessed. The center represents starting point, and black dots represent the ending point. L: left, R: right, F: forward, O: opposite. n = 3 for C‐F. n = 40 for G. Data are expressed as means ± S.E.M. Statistical analyses were performed using one‐way or two‐way ANOVA test with Student's *t* test for C‐E and F. *P < 0.05, **P < 0.01, ***P < 0.001, N.S. not significant.

Molecular docking using AutoDock Vina predicted the binding mode of liraglutide and Myo1c. Liraglutide may directly interact with Myo1c via arginine at position 93 (Arg 93) of Myo1c with the lowest binding energy (−8.3 kcal mol^−1^) (**Figure** **5**A). We generated a Myo1c mutant (Myo1c‐MUT, Arg93 to Gly93) with liraglutide‐binding site mutations and investigated whether this binding site initiated Myo1c/Dock5 signaling and modulated keratinocyte functions. Transfection with the mutant Myo1c resulted in the loss of its interaction with liraglutide in the biotin pull‐down assay (Figure 5B). In addition, induction of Dock5 expression by liraglutide was abrogated in Myo1c‐MUT overexpressed cells (Figure 5C). Moreover, liraglutide did not promote the proliferation, adhesion, or migration of Myo1c‐MUT keratinocytes (Figure 5D–G). These data suggest that arginine 93 of Myo1c is necessary for the regulatory effects of liraglutide on Dock5 and its corresponding keratinocyte functions. Taken together, our findings demonstrate that liraglutide binds to Myo1c to initiate Dock5 signaling and subsequently facilitates keratinocyte behavior, resulting in the promotion of diabetic wound healing.

## 3 Discussion

There is still a lack of clinical guideline recommendations for diabetic wounds and ulcer complications despite the dazzling array of antidiabetic drugs available in the market. Therefore, it is crucial to identify hypoglycemic agents that promote the natural healing process of wounds, in addition to blood sugar control, to improve the quality of life of patients with DFU and reduce national expenditure. In this study, we demonstrated that liraglutide enhanced keratinocyte function and accelerated diabetic wound closure via the effector protein, Dock5. Mechanistic studies revealed that liraglutide binds to Myo1c to stabilize its protein and subsequently upregulates Dock5 expression by directly targeting its promoter. These findings clarify the role and mechanism of liraglutide in diabetic wounds, suggesting that liraglutide may have additional benefits in the treatment of DFU, and provide evidence for its clinical use.

Wound healing is an orderly physiological process consisting of four overlapping steps: hemostasis, inflammation, proliferation, and tissue remodeling.^[^ ^30^ ^]^ Re‐epithelialization is crucial for rebuilding the epidermal barrier and is mainly derived from biological events such as keratinocyte proliferation and migration.^[^ ^15^ ^]^ In addition, the ability of basal keratinocytes to adhere to the basement membrane through the ECM supports cell motility, thereby promoting healing.^[^ ^16^, ^17^ ^]^ However, the functions of epidermal keratinocytes are impaired in diabetes, which contributes to dysregulated and stalled cell phenotypes and healing processes.^[^ ^31^, ^32^ ^]^ Here we show that the clinical antidiabetic drug liraglutide improves skin repair and promotes the proliferation, migration, and adhesion of keratinocytes. In addition to keratinocytes, other cells such as immune cells are activated and recruited to wounds during the healing process, and the types of immune cells vary at different stages of healing. During the proliferation stage, which was the focus of the current study, M2 macrophages and Th2 lymphocytes were the primary immune cells. M2 macrophages release TGF‐β, VEGF‐A, and other cytokines that trigger the transition from fibroblasts to myofibroblasts. Myofibroblasts release MMPs, which degrade the provisional matrix and synthesize ECM components. Th2 lymphocytes produce cytokines such as IL‐4, IL‐5, IL‐13, and IL‐10, which promote collagen production by fibroblasts.^[^ ^33^ ^]^ However, in diabetic wounds, the transformation of M1 to M2 macrophages is impaired, leading to persistent activation of pro‐inflammatory M1 macrophages and impaired wound healing.^[^ ^34^ ^]^ It has been reported that liraglutide decreased the number of Th2 cells and attenuated respiratory syncytial virus‐induced immunopathology.^[^ ^35^ ^]^ Moreover, previous researches suggested that liraglutide may possess anti‐inflammatory properties in the coronary arteries of type 2 diabetes.^[^ ^36^ ^]^ In our study, we also found the inflammatory response was alerted in liraglutide treated diabetic wounds at proliferation stage, suggesting that liraglutide‐regulated diabetic wound healing may also involve the modulation of immune cells.

As a GLP‐1 analog/GLP‐1R agonist, liraglutide is primarily used to treat type 2 diabetes mellitus because of its ability to lower blood glucose and body weight. Later clinical evidence suggested that it exerts not only effects on glucose and obesity treatment, but also protects against diabetic complications, such as diabetic cardiovascular and renal diseases.^[^ ^37^, ^38^ ^]^ In addition, its effects on improving DFU were first reported in the LEADER study, a randomized, double‐blind, multicenter clinical trial. In this study, there was a significant reduction in the risk of amputations related to DFU in the liraglutide group compared to that in the placebo group.^[^ ^10^ ^]^ Following, STARDUST trial, which examined the effects of liraglutide on peripheral artery disease (PAD), a main determinant of the onset and prognosis of DFU in patients with diabetes, reported that liraglutide increased peripheral perfusion during 6 months of treatment.^[^ ^39^ ^]^ Thus, liraglutide shows a strong potential for enhancing DFU treatment. Regarding the limitations of liraglutide in treating DFU, the biggest drawback is the limited direct clinical evidence. Studies, including the LEADER trial, have primarily set diabetic cardiovascular complications as their primary outcome. Direct evidence specifically targeting DFU treatment is limited and requires further research. The adverse effects of liraglutide, such as nausea, vomiting, and diarrhea, may also affect patient adherence to treatment. The delivery method is another important aspect of the therapeutic effects of DFU. In recent decades, several newly generated delivery tools have been extensively studied for wound healing owing to their antibacterial activity, angiogenesis promotion, skin appendage regeneration, and immunoregulatory properties. For example, bioceramic materials with ion‐mediated multifunctionality can enhance wound healing by providing controlled release of therapeutic ions at the wound site, ensuring prolonged and targeted therapeutic effects.^[^ ^40^ ^]^ Immunomodulatory nanosystems with adjustable sizes, variable charges, and high surface‐to‐volume ratios enhance the encapsulation of bioactive factors, offering superior pharmacokinetic and pharmacodynamic profiles for wound healing.^[^ ^41^ ^]^ Photothermal‐enhanced in situ supramolecular hydrogel promotes ·OH generation, creating a gel with antimicrobial properties at the wound site.^[^ ^42^ ^]^

In our study, liraglutide‐treated mice showed a decreasing trend without significant changes in fasting glucose levels across all measured time points, indicating the benefits of liraglutide on wound healing may not mainly via its hypoglycemic effect. These results are consistent with previously reported liraglutide treatments in KK/Ta‐Akita mice, STZ rats, and short‐term db/db mice.^[^ ^20^, ^43^, ^44^, ^45^ ^]^ Possible reasons for this could be related to the models of diabetes, duration and dosage of treatment, wound generation, and absorption of the drug at the wound edges, which are worthy of future research. In addition, the primary aim of this study was to demonstrate that liraglutide directly affects keratinocytes at the wound edge, thereby promoting wound healing. However, it is plausible that liraglutide may affect food intake during the 11‐day treatment period, which could potentially contribute indirectly to wound healing and cannot be entirely ruled out. Consequently, we intend to examine this aspect further in future studies. Given the reported locations of GLP‐1R expression in the brain, stomach, lung, endothelial cells, smooth muscle cells, and specific atrial and ventricular cardiomyocytes,^[^ ^46^ ^]^ the functional effects of liraglutide are often considered to be GLP‐1R‐dependent. However, our study found that liraglutide promoted keratinocyte function, which contributed to skin repair in diabetic mice and might not be mainly mediated by GLP‐1R signaling. Similar studies of liraglutide were reported on reduction of platelet activation reduction,^[^ ^47^ ^]^ attenuation of hepatic inflammation and fibrosis^[^ ^48^ ^]^ and protection against lethal renal ischemia‐reperfusion injury.^[^ ^49^ ^]^ It is interesting to note that these effects mainly occur in organs with low GLP‐1R expression; therefore, whether other effector proteins mediate the action of liraglutide in these organs is worthy of investigation.

Myo1c is an unconventional class I myosin and actin‐based motor protein that is vital for cellular functions, such as intracellular trafficking, cell adhesion, motility, and maintenance of membrane tension.^[^ ^50^, ^51^, ^52^, ^53^ ^]^ Previous researches suggested a potential target of Myo1c for the treatment of liver fibrosis and podocytopathies.^[^ ^54^, ^55^ ^]^ Myo1c was recently reported to participate in hepatic cell proliferation and the wound‐healing response.^[^ ^54^ ^]^ Here, we identified Myo1c as a mediator of liraglutide‐induced keratinocyte proliferation, migration, and adhesion. We further demonstrated that arginine 93 of Myo1c is the binding site for liraglutide, which stabilizes its protein expression. The augmented Myo1c protein and enrichment of the binding between Myo1c and the Dock5 promoter resulted in the promoted Dock5 transcription, which initiated downstream effects in keratinocytes. This is consistent with the proposed function of Myo1c, which has been shown to interact with the promoter of the target gene as a transcription factor.^[^ ^55^, ^56^, ^57^ ^]^

Dock family members share a high degree of conservation and exhibit overlapping tissue expression. Existing research highlights that cellular processes such as proliferation, migration, and adhesion are influenced by the Dock family of proteins.^[^ ^58^, ^59^, ^60^ ^]^ Dock5 was originally shown to participate in myoblast fusion,^[^ ^61^ ^]^ mast cell degranulation,^[^ ^62^ ^]^ bone resorption^[^ ^63^ ^]^ and cell motility in head and neck squamous cell carcinoma.^[^ ^64^ ^]^ Recently, Dock5 has been found to contribute to the regulation of hepatic insulin activity, glucose homeostasis,^[^ ^65^ ^]^ and podocyte lipid metabolism.^[^ ^66^ ^]^ Our previous study revealed the involvement of Dock5 in the wound‐healing process,^[^ ^24^ ^]^ and this study further expands the role of Dock5 as a downstream target of liraglutide in diabetes.

Collectively, our findings reveal a novel role of liraglutide in promoting keratinocyte function and diabetic wound healing, mainly through modulation of Dock5 by binding to Myo1c, providing an evidence base for the future clinical application of liraglutide in DFU treatment.

## 4 Experimental Section

### Reagents

Insulin and liraglutide were purchased from Selleck chem, Houston, TX, USA. Exendin (9‐39) was purchased from MedChem Express,Shanghai, China. Forskolin was obtained from Abcam, MA, USA. Streptozotocin (STZ) was purchased from Sigma–Aldrich, MO, USA. The following antibodies were used in the article: proliferating cell nuclear antigen (PCNA, Santa Cruz, CA, USA), Collagen 1 (Proteintech, Hubei, China), Vimentin (Santa Cruz, CA, USA), Dock5 (novus and Shanghai Benegene Biotechnology), Myo1c (Abcam, MA, USA,) and β‐Actin (Santa Cruz, CA, USA).

### Cell Culture and Transfection

Human immortalized keratinocytes (HaCaT cells) were obtained from the Institute of Basic Medical Sciences, Chinese Academy of Medicine Science (Beijing, China) and were cultured in DMEM medium (Gibco, ThermoFisher, Shanghai, China) supplemented with 10% fetal bovine serum (Gibco, ThermoFisher, Shanghai, China) and 1% penicillin/streptomycin (HyClone, ThermoFisher, Shanghai, China), under standard humid conditions at 37 °C with 5% CO2. Knockdown of RNA expression in HaCaT cells was achieved by seeding cells in 6‐well plates and transiently transfected with siRNA oligonucleotides at 30 pmol per well using Lipofectamine RNAimax (Invitrogen, CA, USA) according to the manufacturer's recommended guidelines. Dock5‐specific, GLP‐1R‐specific, Myo1c‐specific, and corresponding negative control siRNAs were synthesized by Sangon Biotechnology (Shanghai, China). Overexpression in cells was achieved by seeding cells in 6‐well plates and cells were transiently transfected with 1 µg plasmids per well using Lipofectamine 3000 reagent (Invitrogen, CA, USA) according to the manufacturer's instructions. Dock5, Myo1c, and the control vector plasmids were generated by Sino Biological Inc. (Beijing, China).

### Animal Studies

Male C57BL/6, type 2 diabetes (BKS‐Lepr^em2Cd479/Gpt^, db/db) mice, Dock5^flox/flox^ (Dock5^fl/fl^) and keratin14‐Cre recombinase (Cre) mice were purchased from Gempharmatech Company, Jiangsu, China. Keratinocyte‐specific keratin14‐Cre Dock5^fl/fl^ (cKO) mice were generated by crossing Dock5^fl/fl^ mice with keratin14‐Cre mice. Type 1 diabetes was experimentally induced by treating C57BL/6 or cKO mice by streptozotocin (STZ) treatment. Briefly, C57BL/6 or cKO mice aged 8–12 weeks were intraperitoneally injected with 50 mg kg^−1^ STZ for five consecutive days. Two weeks after STZ injection, fasting blood glucose (FBG) was measured, and mice with FBG higher than 13.9 mmol L^−1^ (250 mg dL^−1^) were considered diabetic. All animal procedures and care were performed in accordance with national guidelines and approved by the Laboratory Animal Welfare and Ethics Committee of the Army Medical University (AMUWEC20235043).

### Skin Wound Models and Treatment

Briefly, the mice were anesthetized, and their backs were shaved. Circular 6 mm full‐thickness cutaneous wounds were generated on either side of the midline of the dorsum using a sterile 6‐mm biopsy punch (HealthLink, Jacksonville, FL). For db/db mice, PBS (PBS group) or liraglutide at 200 µg kg^−1^ (Lira group) was subcutaneously injected around the wound margins of mice after the wounds created once a day, or the GLP‐1R antagonist exendin (9‐39) (Ex‐9) was subcutaneously injected alone (Ex‐9 group) or 30 min before each liraglutide administration (Ex‐9+Lira) at a dose of 200 µg kg^−1^ every day. For STZ‐induced diabetic mice, liraglutide was subcutaneously injected at a dose of 200 µg kg^−1^ around the wound margins after the wounds created once a day (STZ+Lira group), and an equal volume PBS was subcutaneously injected as control (STZ group). The wound area was quantified using the ImageJ software (National Institutes of Health, Bethesda, MD) based on digital images acquired after wounding (day 0) and on days 1, 3, 5, 7, 9, and 11 after injury. The percentage of initial wound closure was calculated using the following formula: final wound area on day X/initial area on day 0 × 100%. The mice were euthanized on the indicated day and 8 mm of wounded perilesional skin was collected for histological, gene and protein expression, and collagen deposition analyses.

### Hematoxylin and Eosin (H&E), Masson's Trichrome and Immunostaining Analysis

The mice skin tissues of all the groups were fixed with 4% paraformaldehyde at least 48 h before ethanol dehydration, and the skin tissues were embedded by paraffin and sectioned to 5 µm in thickness for the observation of re‐epithelialization and collagen fiber deposition by Hematoxylin and eosin (H&E) staining, Collagen 1 and Masson's trichrome staining. Immunofluorescence staining for PCNA, and vimentin was performed to assess cell proliferation and tissue granulation. The percentage of PCNA‐positive cells was calculated by dividing the number of PCNA‐positive cells by the total number (DAPI) of cells in the epithelial tissue. All histological and immunostaining images are representative of multiple experiments.

### Quantitative Real‐Time PCR (qRT‐PCR)

TRIzol reagent (Takara) was used to isolate total RNA from skin tissues and cultured cells, the total RNA was then transcribed into cDNA using a PrimeScript RT Reagent Kit (Takara) following the manufacturer's instructions. qRT‐PCR was performed using the SYBR Premix Ex Taq II (Takara) on an Applied Biosystems 7300 System (Life Technologies). All data were analyzed using the expression of GAPDH as an internal control by the ΔCt method. Primer sequences were listed in Table S2 (Supporting Information).

### cAMP Determination

Intracellular cAMP levels were determined using a cAMP Direct Immunoassay Kit (Abcam, MA, USA), according to the manufacturer's instructions. Briefly, cells were seeded into 6‐well plates following the indicated treatments and lysed in lysis buffer. The cell lysates were added to the wells of an anti‐cAMP‐coated 96‐well plate and incubated with the HRP‐cAMP conjugate at room temperature for 2 h. Subsequently, AbRed working solution was added to each well and incubated at room temperature for 1 h. The fluorescence was measured using a SpectraMax i3x microplate reader (Molecular Devices).

### Cell Proliferation and Adhesion Assay

For the cell proliferation assay, the cells were seeded into 96‐well plates. Cell proliferation was assessed using Cell Counting Kit‐8 assay (CCK‐8, MedChemExpress) at the indicated time points. Absorbance was measured at 450 nm using a SpectraMax i3x microplate reader (Molecular Devices). For the cell adhesion assay, cells were cultured in 96‐well plates following treatment, and subsequently collected and reseeded in two 96‐well plates for an additional 4 h of incubation in a cell incubator. To measure the total cell number, one plate was centrifuged, and the medium was removed. The medium of the other plate was removed after the cells were washed with prewarmed (37 °C) PBS to remove unattached cells, and the adherent cells remained in the plate. Cell numbers were quantified using a CyQUANT Cell Proliferation Assay Kit (Invitrogen, CA, USA).

### Wound Scratching and Live Cell Imaging‐Based Migration Assay

The cells were seeded in a 12‐well plate. Scratches were generated by scraping confluent monolayers of cells using a pipette tip and the cells were cultured in fresh serum‐free medium. Images and videos of cell migration were acquired using an optical microscope (Olympus) and live cell imaging (GE, Delta Vision) at indicated time, respectively. Images were analyzed using ImageJ software (National Institutes of Health) and the Chemotaxis and Migration Tool 2.0 (Ibidi).

### RNA Sequencing Analysis

Total RNA from the wounded skin of db/db and liraglutide‐treated db/db mice was harvested using TRIzol reagent (Takara), quantified using NanoDrop2000, and assayed for integrity and purity using an Agilent 5300 Bioanalyzer (Agilent Technologies). RNA sequencing was performed by Majorbio Biotech (Shanghai, China) using an Illumina NovaSeq 6000 platform.

### Biotin Pull‐Down and Mass Spectrometry Assay

Liraglutide or the control was biotin‐labeled with a Sulfo‐NHS‐LC‐Biotin solution using an EZ‐Link Sulfo‐NHS‐LC‐Biotinylation Kit (Thermo Fisher Scientific) according to the manufacturer's protocol. The biotinylated liraglutide (biotin‐Lira) or biotin‐Con samples were incubated with total HaCaT cellular lysate at 4 °C overnight, followed by 2 h incubation with streptavidin‐coupled Dynabeads (Thermo Fisher Scientific). The dynabeads were then washed in TBST buffer, resuspended in SDS‐PAGE sample loading buffer, and boiled at 100 °C for 10 min for separation of the protein and beads. Subsequently, the samples were loaded and separated on an SDS‐PAGE gel, which was stained with Coomassie and cut for mass spectrometry analysis (Shanghai Applied Protein Technology Inc., Shanghai, China) or western blotting.

### Western Blot and Ubiquitination Assays

The skin and cell samples were lysed using RIPA lysis buffer (Beyotime) containing a protease inhibitor (Beyotime). Protein concentration was determined using bicinchoninic acid (BCA) assay (Beyotime). Proteins were separated by SDS‐PAGE, transferred to PVDF membranes (Millipore) and incubated with primary antibodies. Blots were visualized using a chemiluminescent HRP substrate (Millipore) and the FX5s system (Vilber Lourmat). For the ubiquitination assay, cells were transfected with the indicated plasmids containing hemagglutinin (HA)‐ubiquitin and treated with MG132 (10 µM) (Santa Cruz, CA, USA) to block proteasome degradation 4 h before harvest. Cells were harvested and the lysates were analyzed by immunoblotting with antibodies against the HA epitope.

### Dual‐Luciferase Reporter Assay

A luciferase reporter containing the Dock5 promoter sequence was synthesized by Chongqing Jinmai Biotechnology Co., LTD (Chongqing, China). Cells were cultured in 24‐well plates and co‐transfected with firefly luciferase and TK‐Renilla luciferase (internal control). At 24 h after transfection, cells were treated with the indicated treatments and then harvested, and firefly and Renilla luciferase activities were detected using the Luciferase Kit (Promega) according to the manufacturer's instructions.

### Cleavage Under Targets and Tagmentation (CUT&Tag)

A Hyperactive Universal CUT&Tag Assay Kit (TD904; Vazyme) was used for the CUT&Tag experiment. Briefly, HaCaT cells were harvested, counted, and stained with Concanavalin A beads. The cells were resuspended in antibody buffer, incubated with primary antibody overnight at 4 °C, and then incubated with a secondary antibody. Subsequently, the cells were incubated with CUT&Tag pA/G‐Tn5 transposase after sufficient washing. DNA extraction beads were used to extract DNA, and qRT‐PCR was performed using Dock5 primers targeting the promoter region (Table S3, Supporting Information).

### Molecular Docking

Molecular docking was performed using AutoDock Vina software (The Scripps Research Institute, USA). The crystal structure of human Myo1c (PDB ID: 4BYF) was obtained from the RCSB Protein Data Bank (https://www.rcsb.org). The structure of liraglutide was downloaded from the PubChem database (https://pubchem.ncbi.nlm.nih.gov/). The predicted binding energies of the residues were calculated using the ANCHOR web server (http://structure.pitt.edu/anchor/).

### Statistical Analysis

GraphPad Prism software (version 9.0) was used for the statistical analyses. Data are presented as mean ± standard error of mean (S.E.M.). The sample size (n) of each study was described in the corresponding figure legends. Two tail unpaired Student's *t* test and one‐way or two‐way analysis of variance (ANOVA) followed by Tukey's multiple comparisons test were used to assess the significance of difference between groups. Differences was considered significant when expressed as *p < 0.05, **p < 0.01, and ***p < 0.001.

## Conflict of Interest

The authors declare no conflict of interest.

## Author Contributions

Q.Z. and C.Z. contributed equally to this work. Y.Z., HT.Z. Q.Z. designed the experiments. Q.Z., CL.Z., CJ.K., JR.Z. and QS.H. performed the animal studies. H.Q., YR.W., HW. L., Q.T., and M.W. analyzed and interpreted the data. Q.Z., CL.Z. LL.Z. and X.X. performed the cell experiments and helped with data analysis. Y.Z. and Q.Z. drafted the manuscript. Y.Z., H.Q., and HT.Z. guaranteed this work, had full access to all study data, and took responsibility for the integrity and accuracy of the data.

## Supporting information

Supporting Information

## Data Availability

The data that support the findings of this study are available from the corresponding author upon reasonable request.
