# Supplementary material for: Liraglutide Promotes Diabetic Wound Healing via Myo1c/Dock5
Source: Adv Sci (Weinh). 2024 Aug 19;11(39):2405987. doi: 10.1002/advs.202405987 (PMC11497045; doi:10.1002/advs.202405987)
Supplement: Supplementary file 1 — Supporting Information [file ADVS-11-2405987-s001.docx]

**Supplementary Information**

**Liraglutide Promotes Diabetic Wound Healing via Myo1c/Dock5**

Qian Zhang^1,2,3^†, M.S.; Chunlin Zhang^1,2^†, M.S.; Changjiang Kang^2,4^, M.D.; Jiaran Zhu^2^, M.AGR; Qingshan He^2^, M.D.; Hongwei Li^5^, M.D.; Qiang Tong^2^, M.D.; Min Wang^2^, M.D.; Linlin Zhang^2^, M.D.; Xin Xiong^2^, M.D.; Yuren Wang^2^, M.D.; Hua Qu^2*^, M.D., Ph.D.; Hongting Zheng^2*^, M.D., Ph.D.; Yi Zheng^2*^, M.D., Ph.D.

Author Affiliations:

^1^School of Life Sciences, Chongqing University, Chongqing, China.

^2^Department of Endocrinology, Translational Research of Diabetes Key Laboratory of Chongqing Education Commission of China, the Second Affiliated Hospital of Army Medical University, Chongqing, China.

^3^Department of Pharmacy, the Second Affiliated Hospital of Army Medical University, Chongqing, China.

^4^Department of Laboratory Medicine, Chongqing University Three Gorges Hospital, School of Medicine, Chongqing University, Chongqing, China.

^5^Department of Medicinal Chemistry, Army Medical University, Chongqing, China.

Authorship note: † These authors contributed equally to this work.

*Correspondence addressed to:

Yi Zheng, M.D., Ph.D., Email: cecilia.zy@163.com, Hongting Zheng, M.D., Ph.D., Email: fnf7703@hotmail.com, and Hua Qu, M.D., Ph.D., Email: quhuahua120@163.com.

Department of Endocrinology, Translational Research of Diabetes Key Laboratory of Chongqing Education Commission of China, the Second Affiliated Hospital of Army Medical University, Chongqing, 400037, China.

Phone: +8602368755709, Fax: +8602368755707


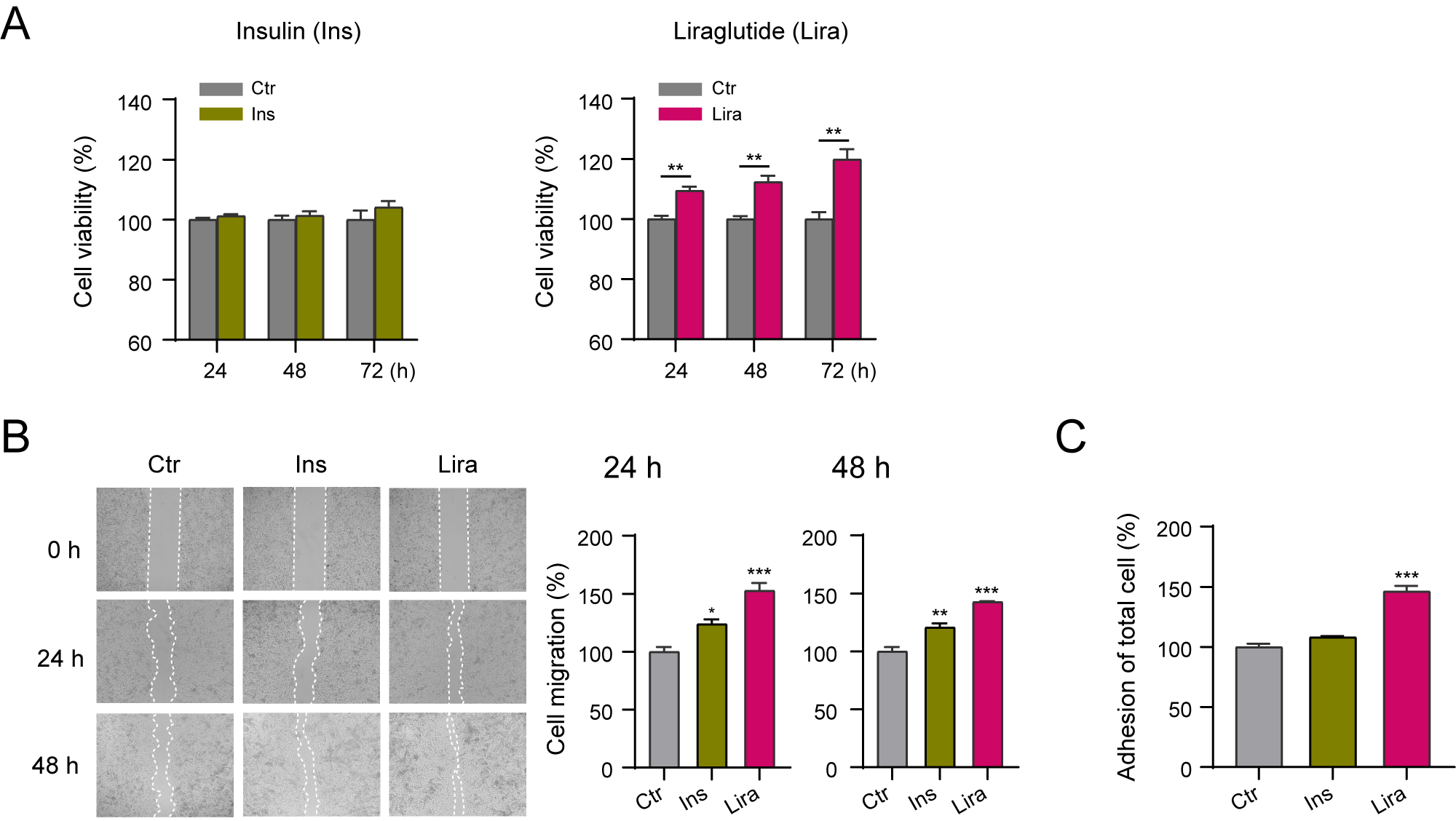


**Figure S1. Effect of insulin and liraglutide on functions of keratinocytes.** (**A**) Cell proliferation was evaluated by Cell Counting Kit 8 assay. (**B**) The migratory ability of keratinocytes was evaluated with wound scratching assays. (**C**) Cell adhesion was determined using the CyQUANT Assay Kit. n = 3 for A-C. Data are expressed as means ± S.E.M. Statistical analyses were performed using Student’s *t* test for A, and two-way or one-way ANOVA test for B and C. **P* < 0.05, ***P* < 0.01, ****P* < 0.001.

**
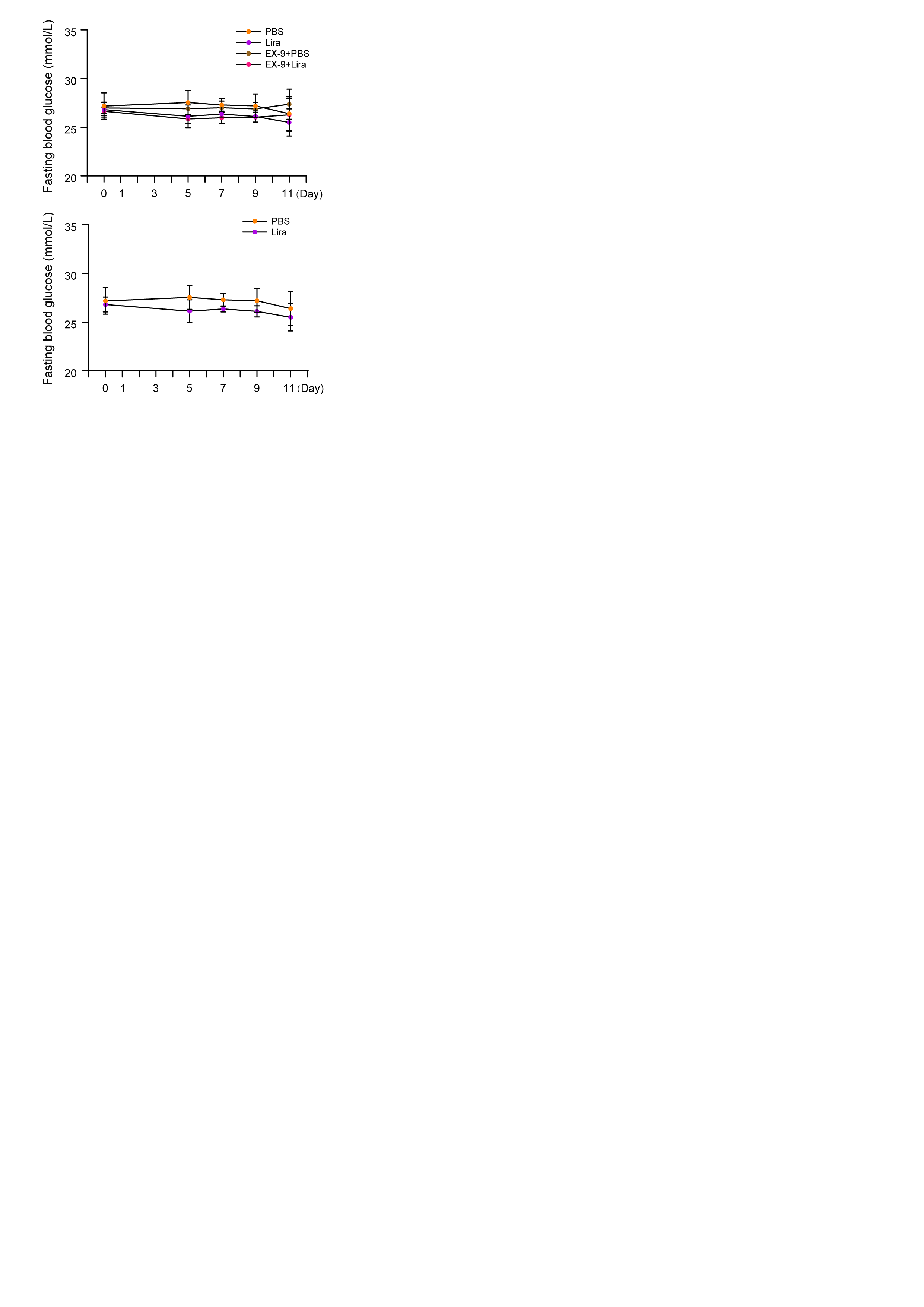
**

**Figure S2. Fasting blood glucose (mmol/L) was measured at day 0, 5, 7, 9 and 11 after injury.** n = 5 mice per group. Data are expressed as means ± S.E.M. Statistical analyses were performed using two-way ANOVA test.

**
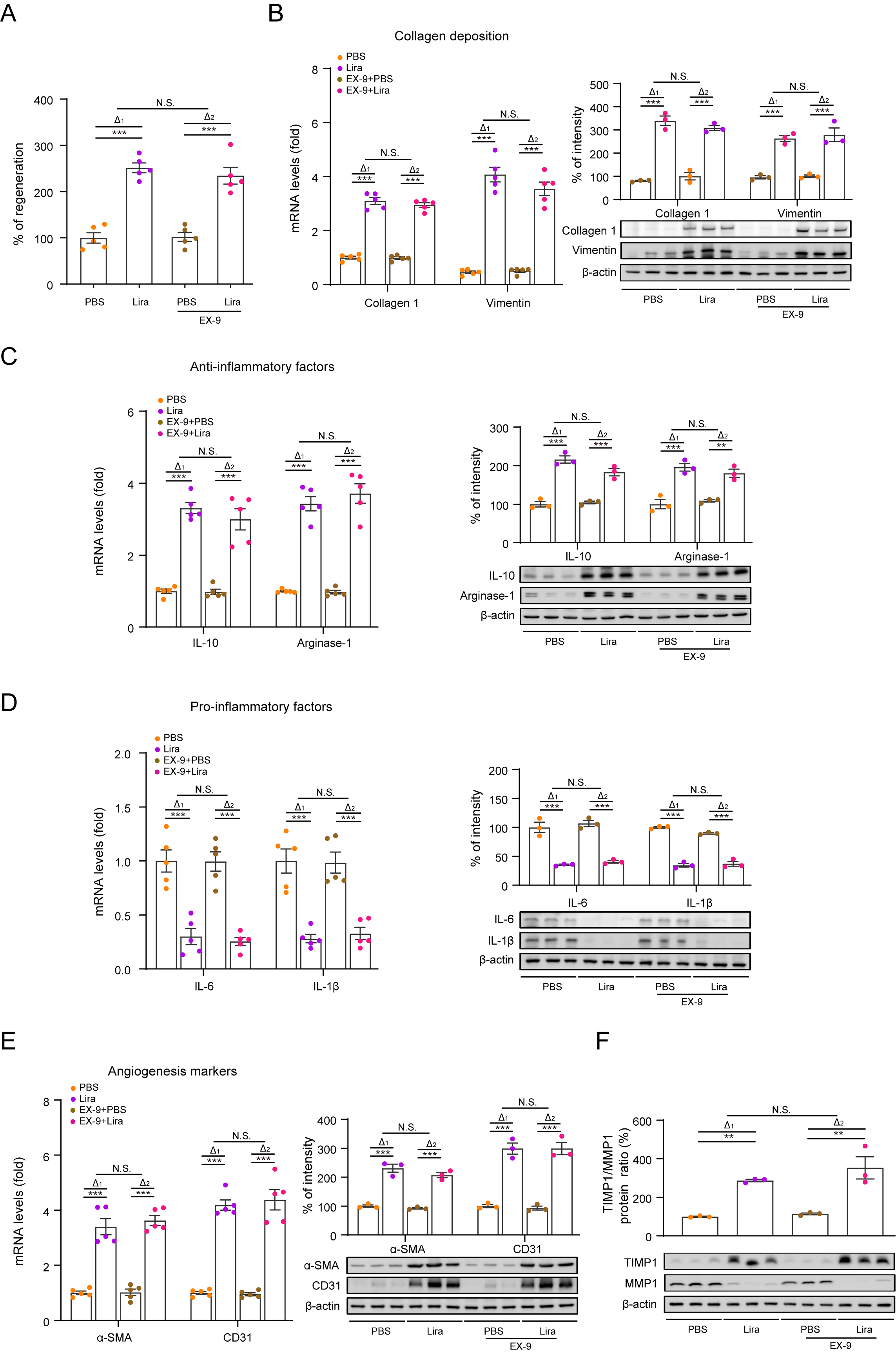
Figure S3. Classical documentation for wound healing including collagen deposition, angiogenesis and inflammatory response is measured in liraglutide treated diabetic mice (related to Figure 1).** (**A**) Quantification of skin tissue thickness (the ratio of regenerated tissue thickness to the original skin tissue thickness) related to H&E staining in Figure 1D. (**B-E**) The mRNA and protein expression of indicated genes related collagen deposition, inflammatory response and angiogenesis. (**F**) TIMP1/MMP1 protein ratio. n = 5 mice per group for A-F. Data are expressed as means ± S.E.M. Statistical analyses were performed using one-way or two-way ANOVA test with Student’s *t* test for A-F. ***P* < 0.01, ****P* < 0.001, N.S. not significant.


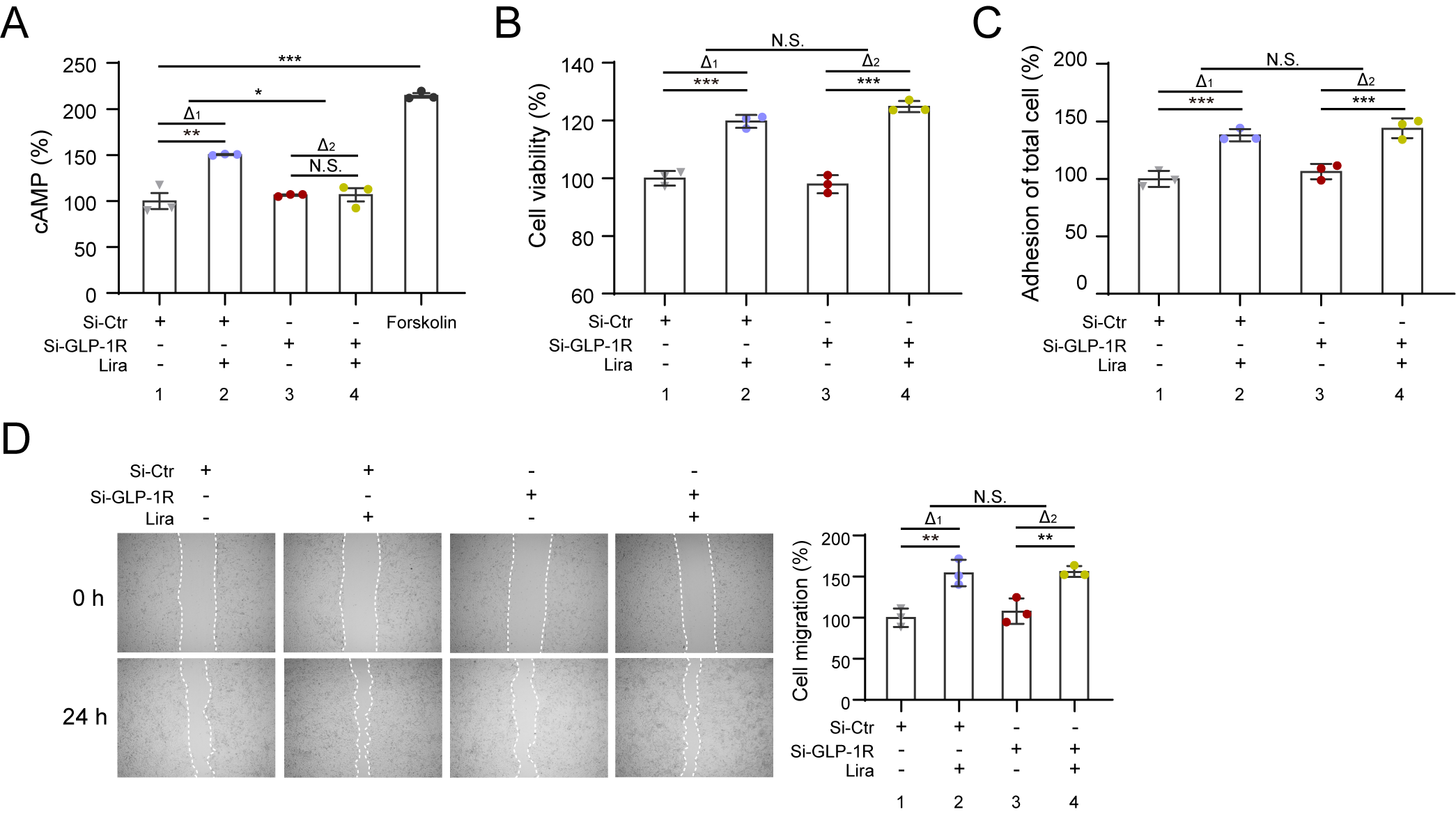
**Figure S4.** **GLP-1R knockdown has little influence on the proliferation, adhesion and migration induced by** **liraglutide in** **keratinocytes.** siRNA for GLP-1R knockdown (si-GLP-1R) or its control (si-Ctr) was transfected in keratinocytes and treated with or without liraglutide (group 1: si-Ctr cells treated with an equal volume of vehicle; group 2: si-Ctr cells treated with liraglutide; group 3: si-GLP-1R cells treated with an equal volume of vehicle; group 4: si-GLP-1R cells treated with liraglutide). (**A**) Intracellular cAMP levels were determined using a cAMP assessment kit and the percentage of cAMP accumulation was calculated. Forskolin (10 μM) was used as a positive control. (**B**) Cell proliferation was evaluated by Cell Counting Kit 8 assay. (**C**) Cell adhesion was determined using the CyQUANT Assay Kit. (**D**) The migratory ability of keratinocytes was evaluated with wound scratching assays. n = 3 for A-D. Data are expressed as means ± S.E.M. Statistical analyses were performed using one-way or two-way ANOVA test with Student’s *t* test for A-D. **P* < 0.05, ***P* < 0.01, ****P* < 0.001, N.S. not significant.

**
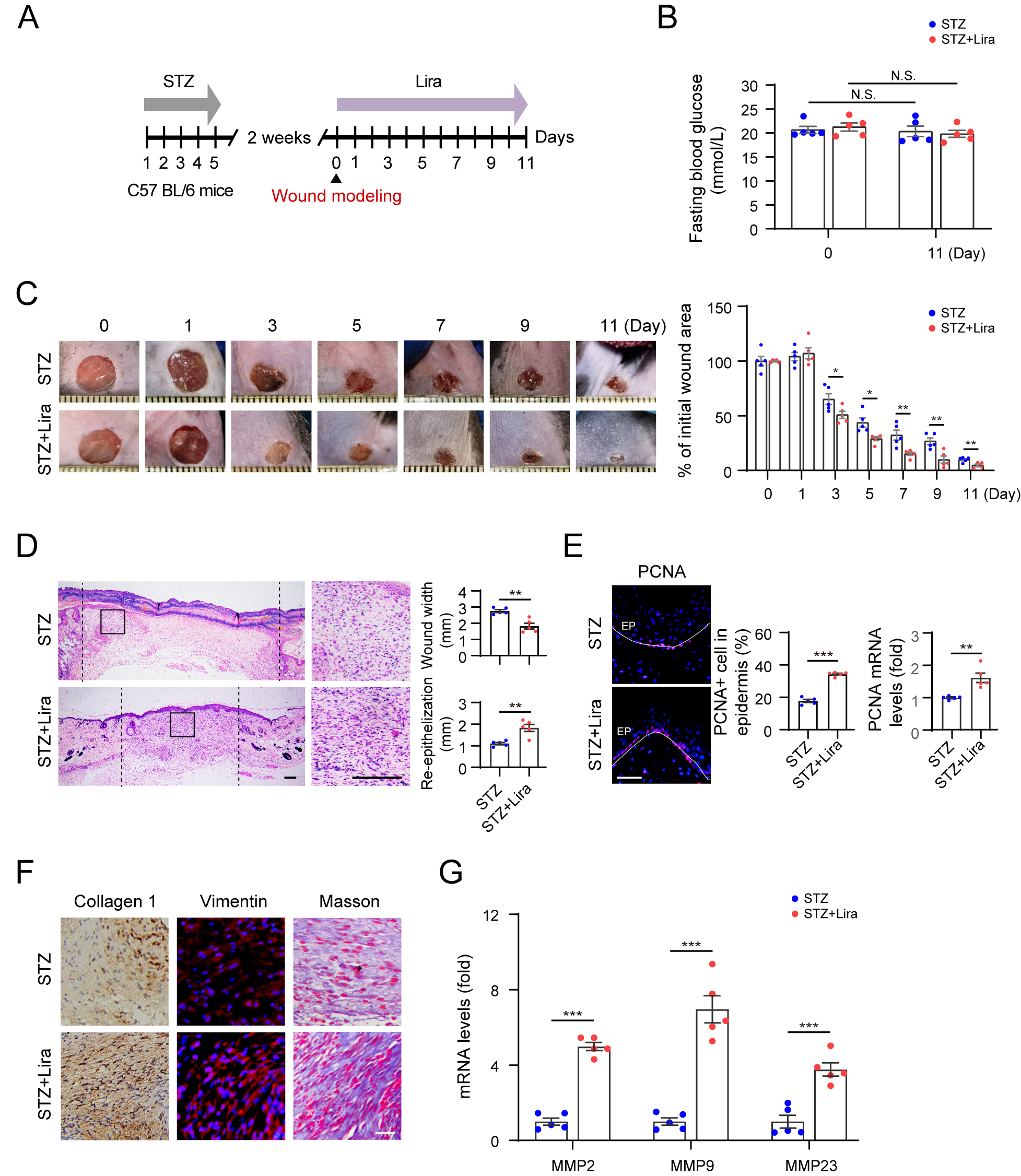
**

**Figure S5. Liraglutide improves wound healing in STZ-induced diabetic mice.** Full thickness wounds were made on the dorsal skin of streptozotocin (STZ)-injected mice for five days (STZ group), and then liraglutide was subcutaneously injected around the wound margins of the STZ mice after the wounds created once a day (STZ+Lira group). (**A**) Schematic for in vivo experiments in STZ-induced diabetic mice. (**B**) Fasting blood glucose (mmol/L) was measured at day 0 and day 11 after injury. (**C**) Images of representative wound (left) and the percentage of the initial wound area (right). Each grid scale represents 1 mm. (**D**) H&E of dorsal skin section was showed and the wound width and re-epithelialization were quantified after 7 days post-injury. Scale bar = 200 μm. (**E**) Representative images and the percentage of PCNA-positive cells in epidermis, and the PCNA mRNA was analyzed by qRT-PCR in wound tissues. EP: epidermis. Scale bar = 50 μm. (**F**) Representative immunostaining images of collagen 1, vimentin and Masson’s trichrome. Scale bar = 50 μm. (**G**) The mRNA levels of indicated genes were analyzed by qRT-PCR in wound tissues. n = 5 mice per group for B-G. Data are expressed as means ± S.E.M. Statistical analyses were performed using Student’s *t* test for B-E and G. **P* < 0.05, ***P* < 0.01, ****P* < 0.001, N.S. not significant.


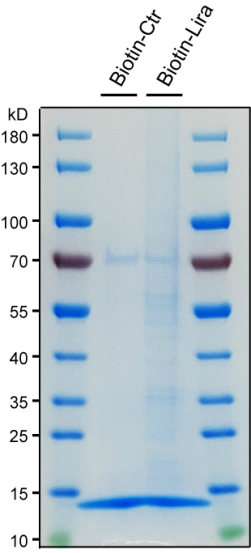


**Figure S6. Coomassie** **staining gel** **for mass spectrometry.** Biotinylated liraglutide (biotin-Lira) and biotin-control (biotin-Ctr) pull-down samples were loaded and separated onto SDS-PAGE gel. The gel was stained by Coomassie and then cut for mass spectrometry.

**
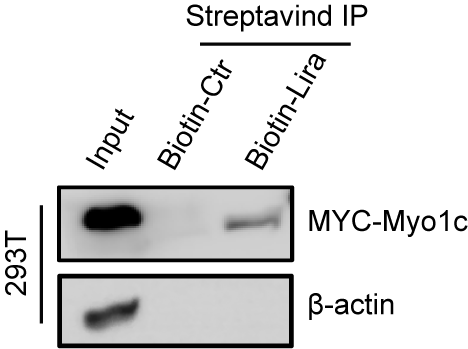
**

**Figure S7. Liraglutide interacts with** **exogenous Myo1c.** The exogenous Myo1c was assessed in HEK 293T cells by biotin-liraglutide pull-down assay, and biotin-azide was used as a control.


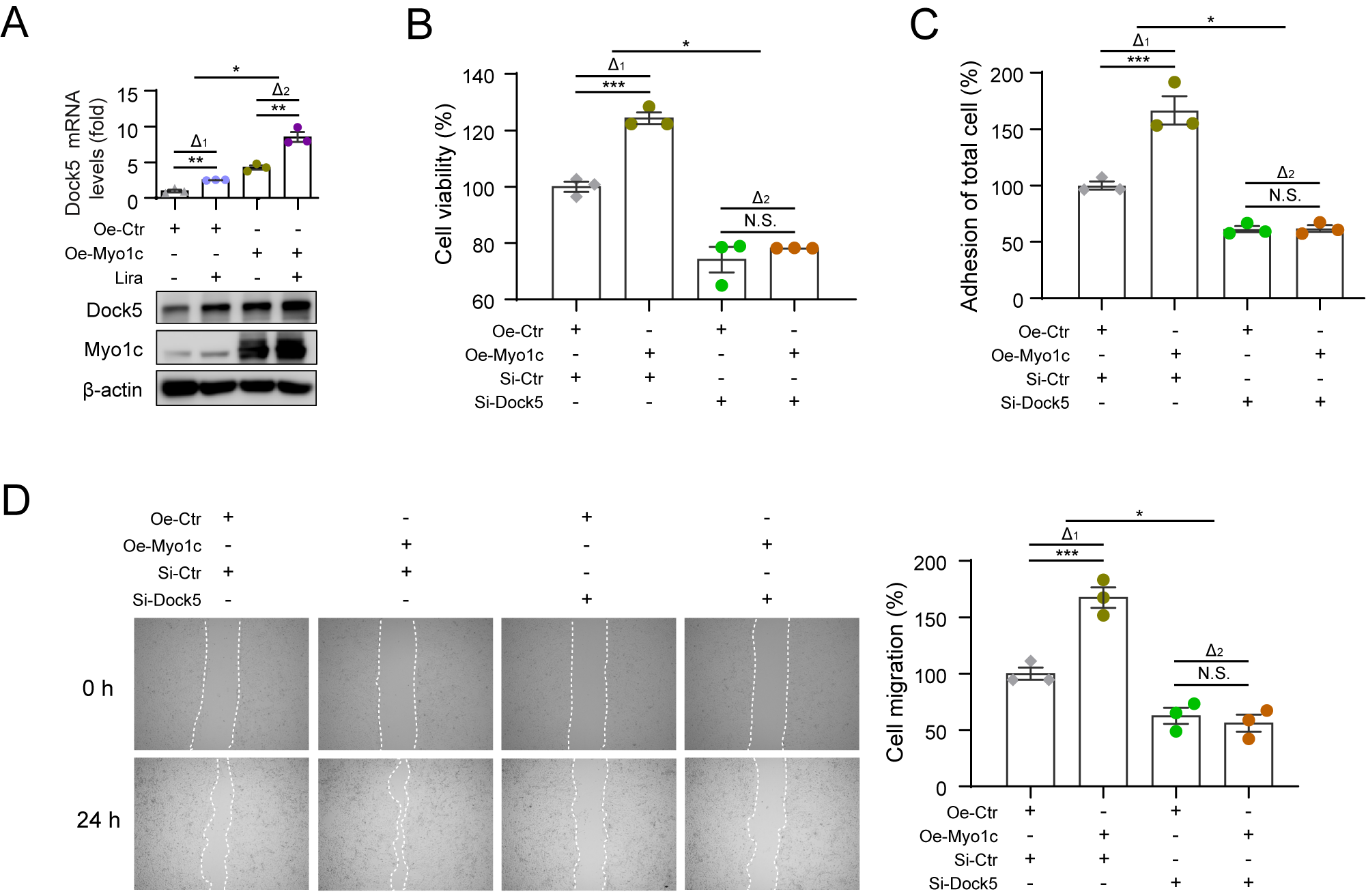


**Figure S8. Myo1c upregulates Dock5 expression and favors keratinocytes functions.** (**A**) The Dock5 mRNA and protein levels were measured in keratinocytes transfected with Myo1c plasmids and treated with or without liraglutide. (**B-D**) Keratinocytes were transfected with Myo1c plasmids and si-Dock5, and cell proliferation (**B**), cell adhesion (**C**) and wound scratching assays (**D**) were evaluated. n = 3 for A-D. Data are expressed as means ± S.E.M. Statistical analysis was performed using one-way ANOVA test with Student’s *t* test for A-D. **P* < 0.05, ***P* < 0.01, ****P* < 0.001, N.S. not significant.

**Table S1. Top 10 terms of Biological Process analysis for upregulated DEGs in wound tissue from db/db mice.**

| **ID** | **Term** | **Ontology** | **Count** | **P_value** | **FDR** | **Enrichment_Factor** | **Fold_Enrichment** |
| --- | --- | --- | --- | --- | --- | --- | --- |
| GO:0030198 | Extracellular_matrix_organization | Biological Process | 63 | 3.32162E-26 | 8.9875E-23 | 3.77697842 | 4.78151490 |
| GO:0007155 | Cell_adhesion | Biological Process | 122 | 3.47746E-26 | 8.9875E-23 | 7.31414868 | 2.83956208 |
| GO:0030199 | Collagen_fibril_organization | Biological Process | 30 | 2.50421E-19 | 4.31476E-16 | 1.79856115 | 7.61730513 |
| GO:0001525 | Angiogenesis | Biological Process | 67 | 1.16524E-17 | 1.50578E-14 | 4.01678657 | 3.23757432 |
| GO:0006954 | Inflammatory_response | Biological Process | 77 | 1.45754E-15 | 1.50681E-12 | 4.61630695 | 2.72230272 |
| GO:0007275 | Multicellular_organism_development | Biological Process | 151 | 7.07294E-15 | 6.09334E-12 | 9.05275779 | 1.91354262 |
| GO:0007156 | Homophilic_cell_adhesion_via_plasma_membrane_adhesion_molecules | Biological Process | 44 | 2.50612E-13 | 1.85059E-10 | 2.63788969 | 3.57245706 |
| GO:0030335 | Positive_regulation_of_cell_migration | Biological Process | 56 | 9.99324E-13 | 6.45688E-10 | 3.35731415 | 2.90722426 |
| GO:0016477 | Cell_migration | Biological Process | 61 | 1.73015E-12 | 9.93681E-10 | 3.65707434 | 2.72162499 |
| GO:0001503 | Ossification | Biological Process | 32 | 1.09196E-10 | 5.64434E-08 | 1.91846523 | 3.81950343 |

**Table S2. List of primers for q****RT-PCR.**

| **Target** | **Forward primer (5′-3′)** | **Reverse primer (5′-3′)** |
| --- | --- | --- |
| **m-Dock5** | GAGGGCCTAGGCAGTCTCTT | ACTGGGTCAGCAACCTCAAG |
| **m-GAPDH** | TGAACGGGAAGCTCACTG | TCCACCACCCTGTTGCTG |
| **m-PCNA** | GAAGTTTTCTGCAAGTGGAGAG | CAGGCTCATTCATCTCTATGGT |
| **m-MMP2** | ACCTGAACACTTTCTATGGCTG | CTTCCGCATGGTCTCGATG |
| **m-MMP9** | GGACCCGAAGCGGACATTG | CGTCGTCGAAATGGGCATCT |
| **m-MMP23** | CCACTTCAACCTCACATACAGAG | GGGGAAACATCACTCCACATTC |
| **m-Collagen 1** | TGAACGTGGTGTACAAGGTC | CCATCTTTACCAGGAGAACCAT |
| **m-Vimentin** | ACGAGTACCGGAGACAGGTG | TGACGAGCCATCTCTTCCTT |
| **m-IL-10** | TGTCATCGATTTCTCCCCTGTG | GCCTTGTAGACACCTTGGTCTT |
| **m-Arginase-1** | GAGACGTAGACCCTGGGGAA | TCCATCACCTTGCCAATCCC |
| **m-IL-6** | TCCATCCAGTTGCCTTCTTG | AAGCCTCCGACTTGTGAAGTG |
| **m-IL-1β** | GAAATGCCACCTTTTGACAGTG | TGGATGCTCTCATCAGGACAG |
| **m-α-SMA** | CCCAGACATCAGGGAGTAATGG | TCTATCGGATACTTCAGCGTCA |
| **m-CD31** | AAAGACCCCCAGAACATGGAT | GAGCCTTCCGTTCTCTTGGT |
| **m-Myo1c** | GAAGGCAGCCAAGAGGAAGT | AGAAGGCATTCTCAGGGCAC |
| **h-Dock5** | CTCCTTGCAGATCGGTGACA | TTTAGATCCTGGGGGAACCCT |
| **h-GAPDH** | GGGTGTGAACCACGAGAAAT | CCTTCCACAATGCCAAAGTT |
| **h-GLP-1R** | CAGCGCTCCCTGACTGAG | CAGGCGTATTCATCGAAGGT |
| **h-Myo1c** | AGTATGCGGTGCCTGTTGTGAA | CGTGATGCTGCCCTGGTTGAT |

**Table S3. Primers for CUT&Tag qRT-PCR.**

Dock5 promoter amplification primers

|  | **Forward primer (5′-3′)** | **Reverse primer (5′-3′)** |
| --- | --- | --- |
| **Dock5** | TCACAGGCTGCAAGGAGGATATAATTT | TCATTTCTTCCTGGAGAGACTTGGAG |
